# Organic sulfur was integral to the Archean sulfur cycle

**DOI:** 10.1038/s41467-019-12396-y

**Published:** 2019-10-07

**Authors:** Mojtaba Fakhraee, Sergei Katsev

**Affiliations:** 1 Large Lakes Observatory, University of Minnesota Duluth, 2205 E. 5th St., Duluth, MN 55812 USA; 2 Department of Physics and Astronomy, University of Minnesota Duluth, Duluth, MN 55812 USA

**Keywords:** Element cycles, Marine chemistry, Sedimentology

## Abstract

The chemistry of the Early Earth is widely inferred from the elemental and isotopic compositions of sulfidic sedimentary rocks, which are presumed to have formed globally through the reduction of seawater sulfate or locally from hydrothermally supplied sulfide. Here we argue that, in the anoxic Archean oceans, pyrite could form in the absence of ambient sulfate from organic sulfur contained within living cells. Sulfides could be produced through mineralization of reduced sulfur compounds or reduction of organic-sourced sulfite. Reactive transport modeling suggests that, for sulfate concentrations up to tens of micromolar, organic sulfur would have supported 20 to 100% of sedimentary pyrite precipitation and up to 75% of microbial sulfur reduction. The results offer an alternative explanation for the low range of δ^34^S in Archean sulfides, and raise a possibility that sulfate scarcity delayed the evolution of dissimilatory sulfate reduction until the initial ocean oxygenation around 2.7 Ga.

## Abstract (web summary)

Marine chemistry during the Early Earth (over 2.7 billion years ago) is commonly inferred to have been inorganically sulfate-reducing. Here, the authors argue that organic sulfur cycling may have played a previously unrecognized, yet important, role in the formation of ancient Archean marine sulfides.

## Introduction

Since the beginning of Life, sulfur cycled between the geosphere and biosphere as an essential component of all living matter. Organic S-bearing molecules, such as amino acids cysteine and methionine, have been ubiquitous throughout the planet’s history^1,2^ and even have been detected on Mars^3^. In modern oceans, however, the petagram inventory^4^ of dissolved organic sulfur and the estimated 0.2–0.4 Pg of particulate organic sulfur^4^ are dwarfed by the 10^9^ Pg of inorganic sulfate, which at 28 mM is the second most abundant anion in seawater. At these concentrations, the geologically important cycling of sulfur through sulfate reduction, precipitation of pyrite, and reoxidation of sulfides is carried out overwhelmingly by inorganic sulfur. Abundant sulfate, however, was rare through most of the Earth’s history. Proterozoic oceans were likely characterized by sub-mM to low mM concentrations^5,6^, and low-sulfate conditions returned episodically throughout the Phanerozoic^7^. In the Archean, before the beginning of ocean oxygenation 2.7–2.5 billion years ago^8,9^, marine sulfate was scarce in coastal and surface pelagic ocean, at no more than tens of µM^9,10^, and likely absent in ferruginous deep waters. The cycling of sulfur under these conditions was very different from the one in the modern ocean, and freshwater systems, particularly stratified lakes, are commonly used as better analogs^10^. Recent work^11^ has demonstrated that in low-sulfate lakes (<100 µM), mineralization of organic sulfur (OS) supplies a significant portion of the substrates for microbial sulfate reduction^12^, and a significant fraction of sulfide is traceable to an organic source^13,14^. In the well-oxygenated sediments of oligotrophic Lake Superior^11^, for example, mineralization of the settled particulate organic sulfur causes accumulation of sulfate in the upper oxidized layer, often in excess of the water column concentrations, and supports over 80% of sulfate reduction in the deeper anoxic sediment. Paradoxically, the organic component has not been considered in reconstructions of the Early Earth sulfur cycling. The histories of atmospheric oxygen and oceanic sulfate are widely inferred from the records of sulfur isotopes preserved in pyrites^9,15^, but non-hydrothermal pyrite formation was considered only from seawater sulfate^10,16^ or elemental sulfur^17^.

Here, we argue that organic sulfur must have been a significant component of the early biogeochemical cycling, and its mineralization provided at least two major pathways to pyrite, which could operate even in sulfate-free environments. The effects of these processes on the geochemical and isotopic compositions of sedimentary sulfides are consistent with available evidence and warrant a re-evaluation of the presently accepted interpretations of the geochemical and isotopic proxies.

## Results

### Arguments for the significance of organic sulfur

Sulfur makes up about 1% of dry weight of aquatic organisms^18^. It occurs at lower oxidation states in proteins such as amino acids cysteine and methionine, in coenzymes (e.g., coenzyme A, biotin, thiamine), as iron-sulfur clusters in metalloproteins, and in bridging ligands (e.g., in cytochrome c oxidase)^2^. Higher oxidation state compounds, such as sulfonates R–SO_3_–H, sulfones R–SO_2_–R, and organo-sulfates, can be found in lipids (e.g., Sulfoquinovosyl diacylglycerols) and are components of cell walls and photosynthetic membranes. Molar S:C ratios in modern plankton^18^ typically range between 0.003 and 0.01, with freshwater values^19^ being more varied than in marine environments because of a wider range of geochemical conditions. Archean S:C ratios likely spanned a similar range, or were even higher if sulfolipids could be used in place of phospholipids^20,21^ to alleviate P limitation^22^. The Archean organic sulfur pool was likely dominated by reduced compounds^23^, which are thermodynamically easier to assimilate under anoxic conditions^24^. Assimilation of sulfate requires energy even at the stage of cellular uptake by sulfate-binding proteins^24^, and sulfate (+6) is rare in prokaryotic cells^24^, whereas key molecules contain sulfites (+4) or sulfonates (+4)^25,26^. Reduced sulfur appears in evolutionary key molecules such as methionine, cysteine, cystine, coenzymes M and acetyl CoA, aromatic sulfur and disulfides, and in primitive metabolic processes such as S oxidation in anoxygenic phototrophs. Hydrothermally supplied hydrogen sulfide (+2) in the presence of CO_2_ could form thiols, critical coenzymes, CS_2_ and dimethyldisulfide^1,27,28^.

**Fig. 1:**
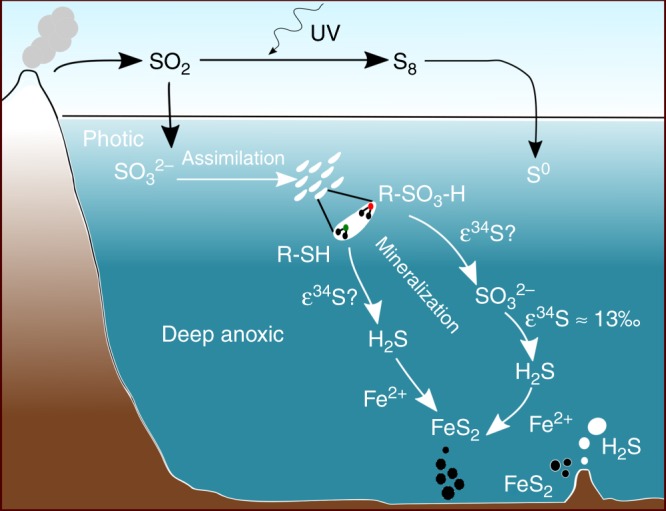
Archean sulfur cycle with contribution from organic sulfur (OS). Mineralization of OS serves as a source of both oxidized (up to +4) and reduced sulfur in the ferruginous deep-water column and sediments. In the Neoarchean, the availability of free oxygen would increase the role of sulfate (+6)

Mineralization of organic sulfur compounds would recycle a significant portion of this organic pool as inorganic sulfur, making it available for processes that in the modern oceans are supported by seawater sulfate. Hydrolysis and mineralization of oxidized organic sulfur (R–SO_3_–H) would generate sulfite (SO_3_^2−^), which in Archean oceans would add to the pool generated by the dissolution of volcanic SO_2_ (Fig. 1). While in the modern environments sulfite is efficiently converted to sulfate by oxidation or disproportionation^29,30^, in an anoxic ancient water column such conversion could take substantially longer, especially if disproportionation^31,32^ was limited by low concentrations or low capacity for microbial catalysis. Mineralization of oxidized organic fractions would supply sulfite throughout the ocean depths that were reached by the inefficient Archean carbon pump^33^, but also, importantly, would generate it in sediments from any exported organic matter. Sulfite is readily utilized by sulfur reducing bacteria for dissimilatory reduction, and thermodynamically provides more energy for cell metabolism than sulfate^34^. Its liberation in a sulfate-depleted ocean would make it available as a substrate for sulfur reducing metabolisms. In a world that lacked a strong oxidant like molecular oxygen, the redox cycling of sulfur could potentially rely on sulfite as the dominant oxidized species. This possibility is supported by genetic evidence that points to the evolution of sulfite reducing metabolisms as early as 3.7 Ga, while genes for sulfate reduction appear later^23,35^. Some Archaea are known to reduce sulfite while not being able to reduce sulfate, and some auxotroph bacteria are known to utilize sulfonate OS directly^4,36^.

Mineralization of the reduced organic S pool (R-SH) would provide an even more important input of inorganic sulfur. Such mineralization generates hydrogen sulfide, which under ferruginous Archean conditions would react with dissolved iron (Fe^2+^) to form iron sulfides, bypassing the traditionally assumed pathway of sulfate reduction.

The geochemical fluxes and transformation rates sustained by the mineralization of organic sulfur must have been quantitatively significant. The inventory and cycling rates of organic matter are poorly constrained for the Archean, but assuming, as an order of magnitude estimate^33,37^, that respiration in the anoxic Archean water column was 5% of the value found in the modern ocean below the photic zone^38^, mineralization rates were ~100 Tmol C year^−1^, regenerating 0.3–1 Tmol S year^−1^ of inorganic sulfur. This is higher than the estimated flux of sulfur from hydrothermal settings^39^ (0.2–0.5 Tmol S year^−1^) and comparable to the estimated Archean pyrite burial flux^40,41^ (~0.1–1 Tmol S year^−1^). Through reduction of oxidized OS and direct release of hydrogen sulfide from reduced OS, followed by a nearly quantitative conversion to pyrite under ferruginous conditions, this re-mineralization could generate a significant portion of the pyrite that was eventually preserved in Archean sediments. Like sulfate reduction, pyrite precipitation likely occurred primarily in organic rich coastal regions, including microbial mats^42^, where the contributions from organic sulfur could have been high. For a concentration of total inorganic sulfur in the ocean water on the order of 10 μM^10^, mineralization at the rate of 0.3–1 Tmol S year^−1^ implies that the entire oceanic pool of sulfur could cycle through the organic pool in under 10,000 years. In surface sediments (e.g., at ~0.1% organic carbon content and the S:C ratio of 0.003), the abundance of organic sulfur (~100 μmol S per liter of sediment, assuming typical porosity and bulk sediment density) would compete with the low μM availability^10^ of inorganic S from the overlying water column, and could be the only source of reactive sulfur in deeper sediment.

Generation of sulfite and dissolved sulfide from organic S within the sediments and water column radically changes the picture of the Archean sulfur cycling. Traditional view and previous numerical models^9,10,16^ assumed that sulfate was transported from the surface ocean into the deep waters or sediments where it underwent microbial reduction to sulfide, which in the presence of ferrous iron precipitated to eventually form pyrite. In contrast, the oxidized inorganic sulfur compounds produced from the oxidized fraction of OS could support S reduction even when sulfate was absent from ambient water. Likewise, the hydrogen sulfide produced from the reduced OS could generate pyrite even in the absence of sulfate reduction.

### Reaction transport modeling

**Fig. 2:**
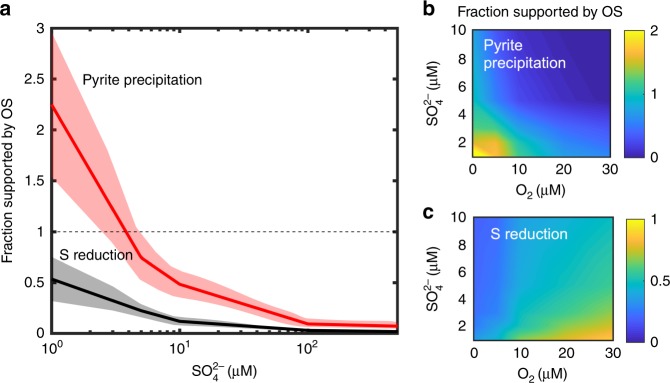
Contribution of organic sulfur to S reduction and pyrite precipitation. **a** As a function of sulfate (sulfite) concentration under anoxic conditions. Shaded bands reflect the corresponding ±1σ ranges obtained in the sensitivity analysis (see Methods section). **b**, **c** The same, in presence of oxygen. The fraction of supported S reduction was calculated as the ratio of the depth integrated rates of oxidized OS mineralization and sediment S reduction. The latter was corrected for the fraction of sulfate reduction supported by elemental S disproportionation and sulfide reoxidation. The fraction of supported pyrite precipitation was calculated as the ratio of the depth integrated rates of reduced OS mineralization and Fe sulfide precipitation. The ratio was corrected for elemental S disproportionation and the fraction of produced hydrogen sulfide that becomes unavailable for precipitation because of aerobic oxidation (see Supplement). Values greater than 1 correspond to a situation where excess hydrogen sulfide diffuses out of the sediment. Sediment was assumed to contain 0.5% of organic carbon by dry weight, with the molar S:C ratio of 0.005

To illustrate the potential role of organic sulfur, we used a vertically resolved diffusion-reaction model, which we adapted from ref. ^11^ and applied to Archean conditions^9^ (see Methods section). For the sake of concreteness, we performed simulations in sediments. While the transport mechanisms in water column may be more varied and three-dimensional, similar arguments should apply, at least qualitatively, to a water column where sulfate and particulate OS reach the anoxic ferruginous deep waters from the surface mixed layer^10^. For a more straightforward comparison with previous models that did not consider sulfite, and to extend the results to potentially oxygenated conditions of the Neoarchean, the model uses sulfate as the oxidized form of inorganic sulfur; this does not change the generality of the argument. Mineralization of reduced OS was assumed in the model to generate hydrogen sulfide (Supplementary Table 1). The ratio of oxidized to reduced OS within organic matter was set to 40%:60% as a reference value and varied in a sensitivity analysis (Supplementary Tables 2 and 3). Similarly to previous work^11^, the model calculated the fraction of the sedimentary sulfate reduction that was supported by OS mineralization, and the fraction of pyrite that was formed through the mineralization of reduced OS (Fig. 2). The latter fraction provided a lower limit for the OS contribution to pyrite formation, as additional pyrite could originate from mineralization of the oxidized OS pool upon its reduction in sediment. These fractions were calculated from the respective ratios of the depth-integrated rates for OS mineralization, sulfate reduction, and pyrite precipitation (Eqs. 1 and 2). As the presence of methane in sediment porewaters may potentially influence the cycling of sulfur, the robustness of the model’s conclusions was tested by including the methane cycle (see Methods section). At the considered low concentrations of sulfate, this modified the inferred ranges for the OS contributions to pyrite formation and sulfate reduction (Fig. 2) by less than 5%.

Simulations reveal that, for <50 µM of sulfate in Archean seawater^9,10^, between 20 and 100% of all pyrite precipitated in sediment would originate from organic sulfur (Fig. 2, Supplementary Figs. 1 and 2). For <10 µM, a significant fraction of pyrite may form from OS even at low organic S:C ratios and for organic carbon concentrations as low as 0.1% (Supplementary Fig. 3). Mineralization of oxidized OS supports between 5 and 75% of the total sedimentary S reduction. The OS contributions remain significant for sulfate concentrations up to >100 μM (Fig. 2, Supplementary Fig. 1), at which point the seawater sulfate becomes the dominant source. If these sulfate concentrations were not achieved in the oceans until the later stages of the Great Oxidation Event^9^, the organic component of the sulfur cycle must have remained important throughout the Archean Eon. At low sulfate concentrations, the presence of oxygen enhances the organic sulfur contribution to sulfate reduction (Fig. 2). Though oceans throughout the Archean Eon are thought to have been predominantly anoxic, Neoarchean sediments in shallow coastal regions could have been exposed to concentrations of up to tens of μM^8,9^. Being a more potent electron acceptor, oxygen decreases the sediment demand for seawater sulfate, making the in-sediment generation of oxidized S proportionately more important. The (percentage) contribution of OS to sulfate reduction increases also with the sediment organic matter content, as organic matter supports OS mineralization rates, even though it also stimulates sulfate reduction and the drawdown of sulfate from overlying water (Supplementary Fig. 3). In the Neoarchean, environments with higher oxygen concentrations and higher organic carbon fluxes could be found in oxygenated oases in shallow coastal regions^8,43^ where oxygenic photosynthesis fueled higher primary productivity, sedimentation rates were high, and most pyrite is thought to have originated.

## Discussion

The contribution of organic component to pyrite formation profoundly changes the accepted interpretations of the Archean isotopic signals. Microbial sulfate reduction depletes the sulfide in ^34^S, generating isotopic differences between the seawater sulfate and sediment pyrite ∆^34^S_FeS2_ = δ^34^S_SO4_–δ^34^S_FeS2._ The limited range of ∆^34^S < 10‰ throughout the Archean is viewed as a consequence of low sulfate, which restricted sulfate reduction^10,16^. The ∆^34^S_FeS_ range is further limited by the Rayleigh distillation: the sulfate diffusing downward into the sulfate reduction zone becomes isotopically heavier with depth and the δ^34^S of the produced sulfide trends towards the δ^34^S of the original sulfate. The possibility of pyrite formation from the OS-derived sulfur means that sulfidic rocks do not necessarily record the evidence of these processes but instead reflect a more complex mixture of isotopic influences. Sulfite produced at some depth within the sediment column may be reduced at the same depth, without undergoing Rayleigh distillation^32^. As isotopic fractionations associated with the reduction of sulfite are small^32,44^ (13 ± 7‰) compared with those for sulfate (>30‰), they are consistent with observations of small ∆^34^S. The hydrogen sulfide produced from the more abundant reduced OS compounds would generate solid sulfides, bypassing microbial reduction. Rather than carrying an isotopic signature of redox processes, these sulfides could instead carry the isotopic signal of hydrolysis. The magnitudes of the fractionations during hydrolysis of organic sulfur are not well constrained, but thermodynamic considerations^45^ and laboratory investigations^46^ limit them to less than 17‰, consistent with small observed ∆^34^S. Small isotopic fractionations are similarly consistent with the evidence in modern sediments where care was taken to analyze the hydrolyzable fraction of organic sulfur^47,48^. In particular, depth variations in the isotopic composition of hydrolyzable organic sulfur pool seem to indicate a preferential loss of isotopically light organic sulfur during the early stages of diagenesis^47^. Incorporating these considerations into an isotopic model of sulfur diagenesis (modified from ref. ^11^; Supplementary Fig. 4) suggests that the ∆^34^S_FeS_ values in Archean pyrites could be increased by the contributions from organic sulfur (assuming fractionation during hydrolysis of 15‰) only by a few permil (Supplementary Fig. 5), even when most of the pyrite originates from organic sulfur (Fig. 2a). The ∆^33^S_FeS_ values of pyrite are essentially not affected (<0.5‰) when the ∆^33^S composition of organic sulfur is similar to that of seawater sulfate.

The ambiguity of isotopic interpretations calls for a re-evaluation of the ancient sulfur cycling. Mineralization of S-bearing organic matter provided sulfur-reducing organisms not only with an electron donor in the form of organic carbon, but also with an electron acceptor, while mineralization of the reduced OS could supply hydrogen sulfide directly. As ∆^34^S_FeS_ records are equally consistent with sulfite reduction and formation of pyrite from reduced organic sulfur, neither of which requires sulfate, the isotopic evidence for an early (3.47 Ga) onset of sulfate reduction^49,50^, suggested based on ~10‰ isotopic fractionations^51^, may need to be re-evaluated. Sulfur isotopes are the only reliable tracer for the sulfate reduction metabolism, as preserved cellular structures are not readily identifiable for sulfate-reducers, while molecular fossils (biomarkers) do not seem to survive over geological times^49^. In a low-oxygen world where sulfate was produced in limited quantities^52^ by atmospheric photochemical reactions, and at somewhat greater but uncertain rates by disproportionation of reactive sulfur intermediates^30,52^, dissimilatory sulfate reduction could have become globally competitive for the first time when sulfate concentrations increased in the Neoarchean, following the initial marine oxygenation around 2.7 Ga^9,53^. The observed expansion in ∆^34^S_FeS_ beginning around that time^5,9^ thus may reflect not only a more vigorous redox cycling of sulfur but also increased isotopic fractionations^54^ associated with the expanded range of redox states. Similarly, while anoxygenic phototrophs nearly universally can oxidize sulfide to elemental sulfur^24^, evidence for the evolution of groups capable of completing the oxidation to sulfate seems to appear first around 2.7 Ga^55^. As pyrite could be formed in non-hydrothermal settings from relatively abundant reduced organic sulfur (Fig. 2), its presence may not necessarily indicate active sulfate reduction, allowing a possibility of only trace amounts of sulfate (sulfite) in oceanic seawater. The organic sulfur pathway under such conditions could generate more pyrite than the reduction of seawater sulfate, and the geographic distribution of such pyrite could be broader than for the pyrite formed from hydrothermal H_2_S. The concentrations of dissolved inorganic sulfur in ferruginous oceans thus could have been low enough to make sulfur a co-limiting nutrient, consistent with the approximately similar S and P contents in living cells.

**Fig. 3:**
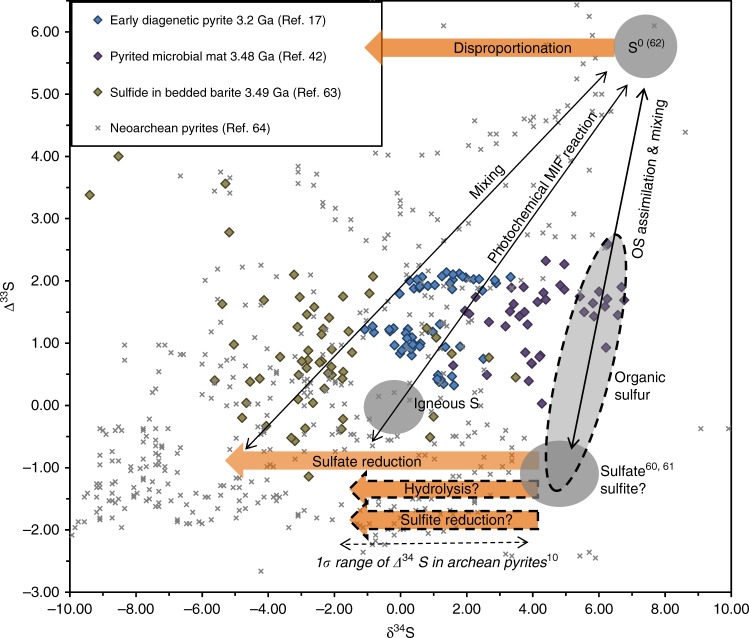
Summary of potential effects of organic sulfur reactions on the isotopic signatures in Archean pyrites. Symbols illustrate the isotopic data from the literature^10,17,42,60–64^ for early (pre-oxygenation) Archean pyrites (blue and purple), Neoarchean pyrites (gray), and sulfide inclusions in sulfate minerals (brown). Gray ovals represent isotopic endmembers; orange arrows denote fractionation-generating microbial reactions. Dashed outlines mark the uncertain contributions and ranges associated with organic sulfur (OS)-specific effects. The poorly constrained Δ^33^S/δ^34^S composition of the OS pool is plotted here along the sulfate-S^0^ mixing line to indicate the possibility of Archean organisms assimilating sulfur from either the seawater sulfate (or sulfite) or atmospherically derived elemental sulfur endmembers. Increased sulfate reduction induced by the Neoarchean ocean oxygenation would be expected to increase Δ^34^S, as sulfate reduction produces stronger isotopic fractionations than OS hydrolysis, reduction of sulfite, or S disproportionation. Errors associated with the data points can be found in the original publications (refs. ^10,17,42,60–64^) from which the data were taken

Resolving the organic sulfur effects in the Archean rock record requires better understanding of the OS pathways and isotopic fractionations than is currently available. Some insight, however, may be obtained from the expected isotopic signatures (Fig. 3). Assuming that Archean microorganisms satisfied their sulfur requirements by assimilating sulfur with the isotopic composition similar to that of seawater sulfate, the Δ^33^S/δ^34^S values in the resultant pyrites should fall closer to the SO_4_–S^0^ mixing line (Fig. 3) than those produced through microbial sulfate reduction, which generates stronger δ^34^S fractionations. Earlier, organic-rich, or sulfate-poor deposits therefore would be expected to have higher pyritic δ^34^S values than those that formed at higher sulfate concentrations, such as in the Neoarchean. While the small amount of Mesoarchean data does not yet allow firm conclusions, this trend seems to be indeed present (Fig. 3). Further insights are likely to be obtained through a combination of laboratory experiments and observations in modern low sulfate environments. In particular, mineral grain-scale signatures^17,42^ of pyrite-forming OS mineralization may be potentially resolved when the OS pathways are better characterized for the conditions of low ambient sulfate, and the associated isotopic fractionations, including those during OS hydrolysis, are better determined.

## Methods

### Geochemical model

The geochemical reaction-transport model for the transformations of organic sulfur and sediment sulfur cycling was adapted from ref. ^11^, with the aspects pertaining to Archean conditions adjusted based on ref. ^9^. The included reactions and their rate formulations are listed in Supplementary Tables 1 and 2. Model parameter values are listed in Supplementary Table 3.

Mineralization of organic carbon and the distributions of iron within the sediment were modeled as described in ref. ^9^, and their control parameters were varied in the sensitivity analysis (see below). Diagenetic formation of elemental sulfur was considered through oxidation of sulfide by iron oxides, which is the pathway that is thermodynamically favorable at low sulfide concentrations^9^. Thiosulfate was not considered because at low sulfide availability aerobic oxidation of sulfide proceeds largely without intermediate redox compounds^24^.

Mineralization of reduced OS was assumed in the model to generate hydrogen sulfide (Supplementary Table 1) whereas mineralization of oxidized organic sulfur (R-O-SO_3_H groups) such as in sulfonates and esters was assumed to generate sulfite or sulfate. As the S:C ratios in the Archean ecosystem are not established, the S:C ratio was varied in the model within a range of literature values for modern marine ecosystems^56^. The S:C ratios may reflect, for example, factors such as organism physiology (specific sulfur requirements for biomass) and environmental conditions such as nutrient limitation. For example, in modern systems, P limitation in plankton is known to stimulate substitution of sulfolipids (and N-based lipids) for phospholipids^20,21^, strengthening the sulfur deposition into sediments.

Organic sulfur was assumed to be delivered into the sediment with particulate organic matter. Similarly to treatment in ref. ^11^, the rate of organic sulfur mineralization, a multi-step process, was simulated as proportional to the rate of organic C mineralization. While enzymatic hydrolysis was considered to be influenced by sulfate availability in soil literature^57^, such inhibition was not shown in modern sediments and thus was not included in the model.

To quantify the fraction (*α*) of sediment S reduction supported by mineralization of organic sulfur, we used the ratio of the depth-integrated rates of sulfate generation and sulfate reduction, corrected for the in-sediment recycling of sulfur by the disproportionation of elemental sulfur and re-oxidation of hydrogen sulfide. To quantify the fraction (*β*) of pyrite precipitation supported by organic sulfur, we used the ratio of the depth-integrated rates of sulfide generation to iron sulfide formation, corrected for the production of sulfide through elemental sulfur disproportionation and reduction of the mineralized oxidized OS. Using R* as the notation for depth-integrated rates of the respective reactions (Supplementary Table 2), the corresponding parameters *α* and *β* are thus defined as:

1 $$\alpha = \frac{{R_{{\mathrm{PSO4}}}^ \ast }}{{(R_{{\mathrm{SR}}}^ \ast + R_{{\mathrm{Assim}}}^ \ast + R_{{\mathrm{CH4}}\_{\mathrm{SO4}}}^ \ast - R_{{\mathrm{SOX}}}^ \ast - 0.25 \times R_{{\mathrm{Disp}}}^ \ast )}}$$

2 $$\beta = \frac{{R_{{\mathrm{PH2S}}}^ \ast - R_{{\mathrm{SOX}}}^ \ast }}{{(R_{{\mathrm{FeS}}}^ \ast - 0.75 \times R_{{\mathrm{Disp}}}^ \ast )}}$$

Values of *α* or *β* greater than 1 would correspond to a situation where mineralization of OS fully supports, respectively, sulfate reduction or pyrite precipitation, with the excess inorganic S fluxing out of the sediment. The fraction of sulfur originated from external inorganic sources, such as seawater sulfate or hydrogen sulfide produced from the reduction of seawater sulfate, are given by 1- *α*, and 1- *β*. At higher oxygen levels, the calculated value of *α* is expected to be a conservative estimate of the OS contribution, as oxidation of organic-sourced reduced sulfur would also replenish the sulfate pool.

### Sensitivity analysis of the geochemical model

The dependence of model’s results on its parameter values was investigated using a sensitivity analysis. The predicted ranges of *α* and *β* (Fig. 2 and Supplementary Fig. 1) were calculated for multiple parameter sets (at least 10 for each sulfate concentration), by randomly and independently selecting model parameter values within their uncertainty ranges (Supplementary Table 3), assuming uniform probability distributions. For parameters whose uncertainty ranges span several orders of magnitude, such as reaction rate constants, the values were selected assuming uniform probability distributions of their logarithms. The analysis revealed that the conclusions presented in the main manuscript are not sensitive to most parameters including pH, initial age of organic matter deposited into sediment, diffusion coefficients, porosity, burial velocity, rate constants for oxidation of sulfide and FeS precipitation, Monod constants for sulfate reduction (*K*_m_), and elemental sulfur disproportionation rate constant. The parameters that affected the values of *α* and *β* most strongly included the sediment organic matter content, sulfur to carbon ratio (S/C), and the proportion of organic sulfur present in oxidized (*f*_SO4_) vs. reduced form (*f*_H2S_). The sensitivity of *α* and *β* to the S/C ratio and organic matter content, as well as the typical sediment geochemical profiles are illustrated in Supplementary Figs. 2–4. As expected, higher S:C ratios elevate the production of inorganic sulfur from organic compounds, which in turn, increases the contributions of organic sulfur in supporting S reduction and pyrite precipitation. Increasing organic matter content also enhances the contribution of OS, as it supports higher OS mineralization rates, even though it also stimulates sulfate reduction, which increases the drawdown of sulfate from overlying water (Supplementary Fig. 3). In difference to previous diagenetic models of sulfur cycling under Archean conditions^9,10^, the sensitivity of the current model was further investigated by considering production of methane through methanogenesis and consumption of methane through aerobic and anaerobic oxidation (AOM) (Supplementary Tables 1 and 2). This affected the values of *α* and *β* by less than 5%, as at the considered low concentrations of sulfate the kinetics of AOM is too slow to reduce a significant fraction of sulfate. Specifically, for the typical range of the reaction rate constant *k*_CH4–SO4_ in Supplementary Table 3, AOM accounted for no more than 4% of the total sedimentary sulfate reduction, and the porewater sulfate profiles were affected imperceptibly.

### Isotopic model

The isotopic model tracked the transformation of sulfur isotopes (^32^S, ^33^S, ^34^S) during diagenesis, including during organic sulfur hydrolysis. Supplementary Fig. 5 illustrates the reactions and isotopic fractionations associated with modeled pathways. Similarly to the previous model^9^, the total rate of sulfate reduction obtained from the geochemical model was partitioned in terms of the individual isotope rates:

3 $$R_{{\mathrm{32SR}}} + R_{{\mathrm{33SR}}} + R_{{\mathrm{34SR}}} = R_{{\mathrm{SR}}}$$

Approximating *R*_32SR_+*R*_34SR_ = 0.9924 *R*_SR_, as the sulfate pool is dominated by ^32^SO_4_ (~96%) and ^34^SO_4_ (~4%), the individual isotope rates were calculated as:

4 $$R_{{\mathrm{32SR}}} = \frac{{0.9924 \times R_{{\mathrm{SR}}}}}{{1 + \frac{\eta }{\alpha }}}$$

5 $$R_{{\mathrm{34SR}}} = \frac{{0.9924 \times R_{{\mathrm{SR}}}}}{{1 + \frac{\alpha }{\eta }}}$$

Here, the fractionation factor

6 $$\alpha = \frac{{R_{{\mathrm{32SR}}}[{\,}^{34}{\mathrm{SO}}_4^{2 - }]}}{{R_{{\mathrm{34SR}}}[{\,}^{32}{\mathrm{SO}}_4^{2 - }]}}$$

describes the preferential use of the lighter isotope, and [^32^SO_4_] and [^34^SO_4_] are concentrations. The concentration ratio *η* = [^34^SO_4_^2−^]: [^32^SO_4_^2−^] is related to the standard *δ*^34^S notation as

7 $$\eta = \frac{{\delta {\,}^{34}{\mathrm{SO}}_4^{2 - } \times \frac{{{\,}^{34}{\mathrm{S}}_{{\mathrm{CDT}}}}}{{{\,}^{32}{\mathrm{S}}_{{\mathrm{CDT}}}}}}}{{1000}} + \frac{{{\,}^{34}{\mathrm{S}}_{{\mathrm{CDT}}}}}{{{\,}^{32}{\mathrm{S}}_{{\mathrm{CDT}}}}}$$

where CDT refers to the ^34^S/^32^S-ratio of the standard troilite, an iron monosulfide from the Canyon Diablo Meteroite. The value of *η* at the beginning of iterations was calculated using the δ^34^SO_4_^2−^ of 12‰. For α, we conservatively imposed a fractionation factor of 30‰ (*α* = 1.030), typical for sulfate reducing bacteria^58^, and decreased it linearly to zero below 6 µM, which reflects sulfate limitation^16^, similarly to previous models^9,10^. Constant fractionation factors (*α*) were used for other reactions: organic sulfur hydrolysis (1.015), sulfide oxidation^16^ (1.005) and elemental sulfur disproportionation^59^ (0.988 for sulfate and 1.007 for sulfide).

The reduction rate for ^33^SO_4_^2−^ was calculated as:

8 $$R_{{\mathrm{33SR}}} = R_{{\mathrm{32SR}}}\frac{{{\mathrm{\eta }}\prime }}{{\alpha \prime }}$$

with the mass-dependent fractionation factor *α*^′ ^= 0.515α. The initial value of *η*^ʹ ^=  [^33^SO_4_^2−^]: [^32^SO_4_^2−^] was calculated using the δ^33^SO_4_^2−^ of 2.5‰:

To considering isotopic fractionation during organic sulfur hydrolysis, the total rate of hydrolysis obtained from the geochemical model was partitioned in terms of the individual rates of oxidized and reduced organic sulfur:

9 $$R_{{\mathrm{32PSO4}}} + R_{{\mathrm{33PSO4}}} + R_{{\mathrm{34PSO4}}} = R_{{\mathrm{PSO4}}}$$

10 $$R_{{\mathrm{32PH2S}}} + R_{{\mathrm{33PH2S}}} + R_{{\mathrm{34PH2S}}} = R_{{\mathrm{PH2S}}}$$

The rates and concentrations for the individual isotopes were calculated iteratively analogously to the process described above for sulfate reduction, using defined fractionation factors. Calculations for other reactions in Supplementary Table 1 were carried out similarly to ref. ^9^.

To calculate vertical concentration profiles, net rates were expressed as follows:

11 $${\mathrm{NR}}{\,}^{32}{\mathrm{SO}}_4^{2 - } = R_{{\mathrm{32SR}}} - R_{{\mathrm{32SOX}}} - R_{{\mathrm{32Disp}}\left( {{\mathrm{SO}}_4} \right)} - R_{{\mathrm{32PSO4}}}$$

12 $${\mathrm{NR}}{\,}^{33}{\mathrm{SO}}_4^{2 - } = R_{{\mathrm{33SR}}} - R_{{\mathrm{33SOX}}} - R_{{\mathrm{33Disp}}\left( {{\mathrm{SO}}_4} \right)} - R_{{\mathrm{33PSO4}}}$$

13 $${\mathrm{NR}}{\,}^{34}{\mathrm{SO}}_4^{2 - } = R_{{\mathrm{34SR}}} - R_{{\mathrm{34SOX}}} - R_{{\mathrm{34Disp}}\left( {{\mathrm{SO}}_4} \right)} - R_{{\mathrm{34PSO4}}}$$

14 $${\mathrm{NRH}}_2{\,}^{32}{\mathrm{S}} = R_{{\mathrm{32SOX}}} + R_{{\mathrm{32FeS}}} + R_{{\mathrm{32S}}^0} + R_{{\mathrm{32FeS}} + {\mathrm{HS}}} - R_{{\mathrm{32SR}}} - R_{{\mathrm{32Disp}}\left( {{\mathrm{H}}_{\mathrm{2S}}} \right)} - R_{{\mathrm{32PH2S}}}$$

15 $${\mathrm{NRH}}_2{\,}^{33}{\mathrm{S}} = R_{{\mathrm{33SOX}}} + R_{{\mathrm{33FeS}}} + R_{{\mathrm{33S}}^{\mathrm{0}}} + R_{{\mathrm{33FeS}} + {\mathrm{HS}}} - R_{{\mathrm{33SR}}} - R_{{\mathrm{33Disp}}\left( {{\mathrm{H}}_{\mathrm{2S}}} \right)} - R_{{\mathrm{33PH2S}}}$$

16 $${\mathrm{NRH}}_2{\,}^{34}{\mathrm{S}} = R_{{\mathrm{34SOX}}} + R_{{\mathrm{34FeS}}} + R_{{\mathrm{34S}}^{\mathrm{0}}} + R_{{\mathrm{34FeS}} + {\mathrm{HS}}} - R_{{\mathrm{34SR}}} - R_{{\mathrm{34Disp}}\left( {{\mathrm{H}}_{\mathrm{2S}}} \right)} - R_{{\mathrm{34PH2S}}}$$

17 $${\mathrm{NR}}{\,}^{32}{\mathrm{S}}^0 = R_{{\mathrm{32S}}^{\mathrm{0}}} - (R_{{\mathrm{32Disp}}\left( {{\mathrm{H}}_{\mathrm{2S}}} \right)} + R_{{\mathrm{32Disp}}\left( {{\mathrm{SO}}_4} \right)}) - R_{{\mathrm{32FeS}} + {\mathrm{S}}}$$

18 $${\mathrm{NR}}{\,}^{33}{\mathrm{S}}^0 = R_{{\mathrm{33S}}^{\mathrm{0}}} - (R_{{\mathrm{33Disp}}\left( {{\mathrm{H}}_{\mathrm{2S}}} \right)} + R_{{\mathrm{33Disp}}\left( {{\mathrm{SO}}_4} \right)}) - R_{{\mathrm{33FeS}} + {\mathrm{S}}}$$

19 $${\mathrm{NR}}{\,}^{{\mathrm{34}}}{\mathrm{S}}^0 = R_{{\mathrm{34S}}^0} - (R_{{\mathrm{34Disp}}\left( {{\mathrm{H}}_{\mathrm{2S}}} \right)} + R_{{\mathrm{34Disp}}\left( {{\mathrm{SO}}_4} \right)}) - R_{{\mathrm{34FeS}} + {\mathrm{S}}}$$

The vertical gradients and concentrations for each isotope were then computed by integrating the diagenetic equations over depth with the rates given by Eqs. (11–19). The concentrations, rates, and isotopic ratio parameters were recalculated iteratively until convergence was reached. The isotopic composition of FeS_2_ was then found by integrating the FeS_2_ precipitation rates for individual isotopes. Based on the diagenetic equations with *D*_*i*_=0:

20 $$\left[ {{\mathrm{Fe}}^{34}{\mathrm{S}}_2} \right] = \frac{{\mathop {\smallint }\nolimits_0^l (R_{{\mathrm{34FeS}} + {\mathrm{S}}} + R_{{\mathrm{34FeS}} + {\mathrm{HS}}}).{\mathrm{d}}x}}{v}$$

21 $$\left[ {{\mathrm{Fe}}^{33}{\mathrm{S}}_2} \right] = \frac{{\mathop {\smallint }\nolimits_0^l (R_{{\mathrm{33FeS}} + {\mathrm{S}}} + R_{{\mathrm{33FeS}} + {\mathrm{HS}}}).{\mathrm{d}}x}}{v}$$

22 $$[{\mathrm{Fe}}{\,}^{32}{\mathrm{S}}_2] = \frac{{\mathop {\smallint }\nolimits_0^l (R_{{\mathrm{32FeS}} + {\mathrm{S}}} + R_{{\mathrm{32FeS}} + {\mathrm{HS}}}).{\mathrm{d}}x}}{v}$$

### Sensitivity analysis of the isotopic model

The sensitivity of the isotopic simulation results to model parameters was performed similarly to the sensitivity analysis of the geochemical model. While the results are mostly similar to the findings described in ref. ^9^, isotopic fractionation during organic sulfur hydrolysis affects the isotopic composition of pyrite. Specifically, preferential mineralization of isotopically light organic sulfur results in an isotopically lighter pyrite. As a result, the isotopic composition of pyrite deviates more from that of the seawater sulfate, resulting in a greater ∆^34^S_FeS2_ (by up to 7‰) (Supplementary Fig. 6). This effect is more pronounced at higher sulfur to carbon ratios where the contribution of organic sulfur hydrolysis is greater (Supplementary Fig. 6). At higher sulfate concentrations, the isotopic composition of pyrite is less affected (Supplementary Fig. 6).

Organic sulfur does not strongly affect the mass independent fractionation of sulfur (MIF-S), characteristic of Archean pyrites. The atmospherically produced MIF-S signal is transmitted from elemental S to pyrite through diagenesis and is influenced by the isotopic composition of iron sulfide and porewater sulfide^9^. Because sulfide can be generated from non–MIF-S sulfate through sulfate reduction and hydrolysis of reduced organic sulfur, its addition to pyrite dilutes the MIF-S signal. While the contribution of organic sulfur slightly increases the amount of sulfide with mass-dependent fractionation, our modeling results indicate that its effect on the ∆^33^S value of pyrite is less than 0.5‰.

## Supplementary information

Supplementary Information

## Data Availability

The authors declare that data supporting the findings of this study are available within this article and its Supplementary Information, and all additional data are available from the corresponding author on reasonable request.
