# Supplementary material for: Organic sulfur was integral to the Archean sulfur cycle
Source: Nat Commun. 2019 Oct 7;10:4556. doi: 10.1038/s41467-019-12396-y (PMC6779745; doi:10.1038/s41467-019-12396-y)
Supplement: Supplementary file 1 — Supplementary Information [file 41467_2019_12396_MOESM1_ESM.pdf]

Supplementary Information

Organic sulfur was integral to the Archean sulfur cycle

Mojtaba Fakhraee* [1], Sergei Katsev* [1, 2]

[1] Large Lakes Observatory, University of Minnesota Duluth, Duluth, MN, USA

[2] Department of Physics and Astronomy, University of Minnesota Duluth, Duluth, MN, USA

* Co-corresponding authors.

Supplementary Figures

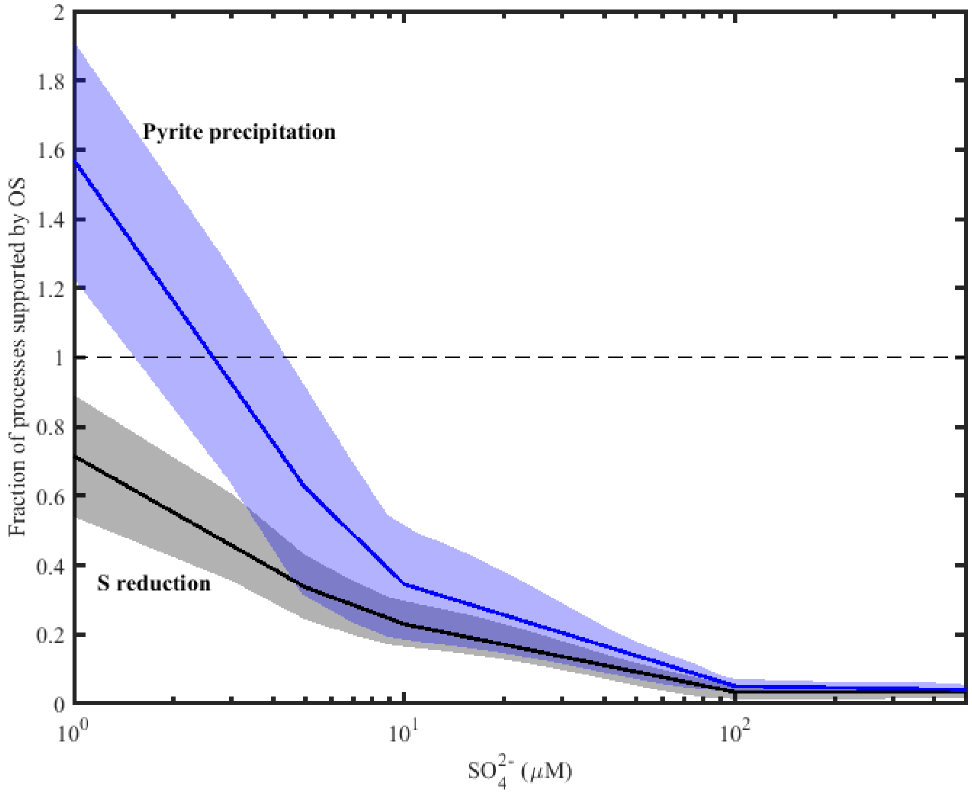

Supplementary Figure 1. Support of sulfate (or sulfite) reduction and iron sulfide formation by organic matter-sourced sulfur (OS) as a function of sulfate (sulfite) concentration under oxic condition ($[O_2]$ = 10 μM). Shaded bands reflect the corresponding ±1σ ranges obtained in the sensitivity analysis.

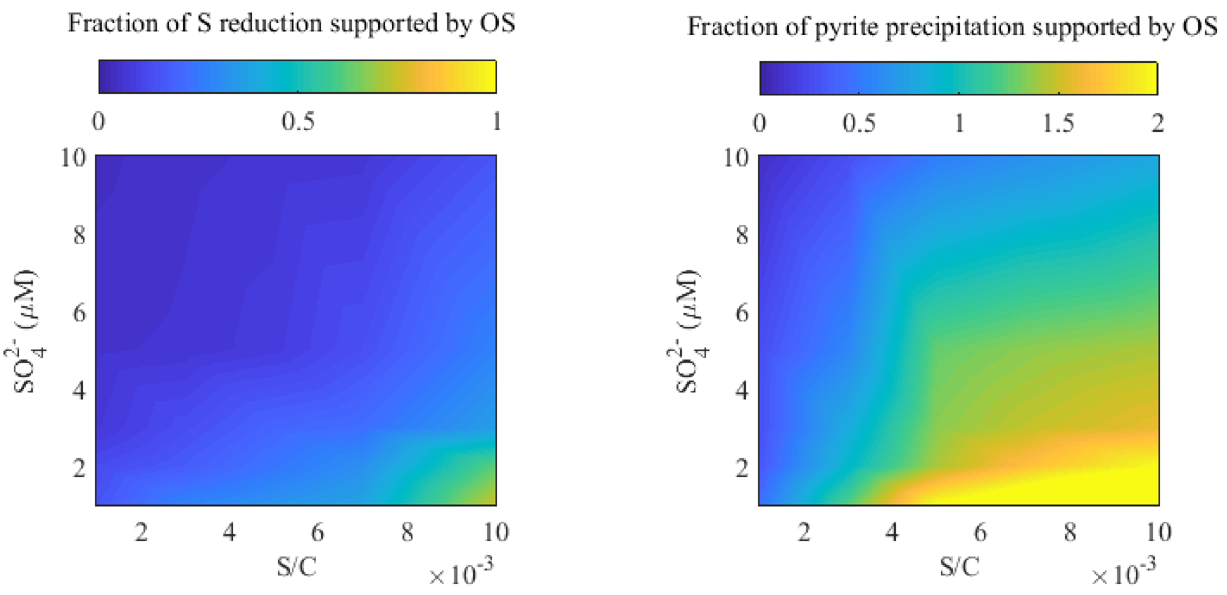

Supplementary Figure 2. Sensitivity of α (left) and β (right) to the S:C ratio in organic matter, under anoxic conditions.

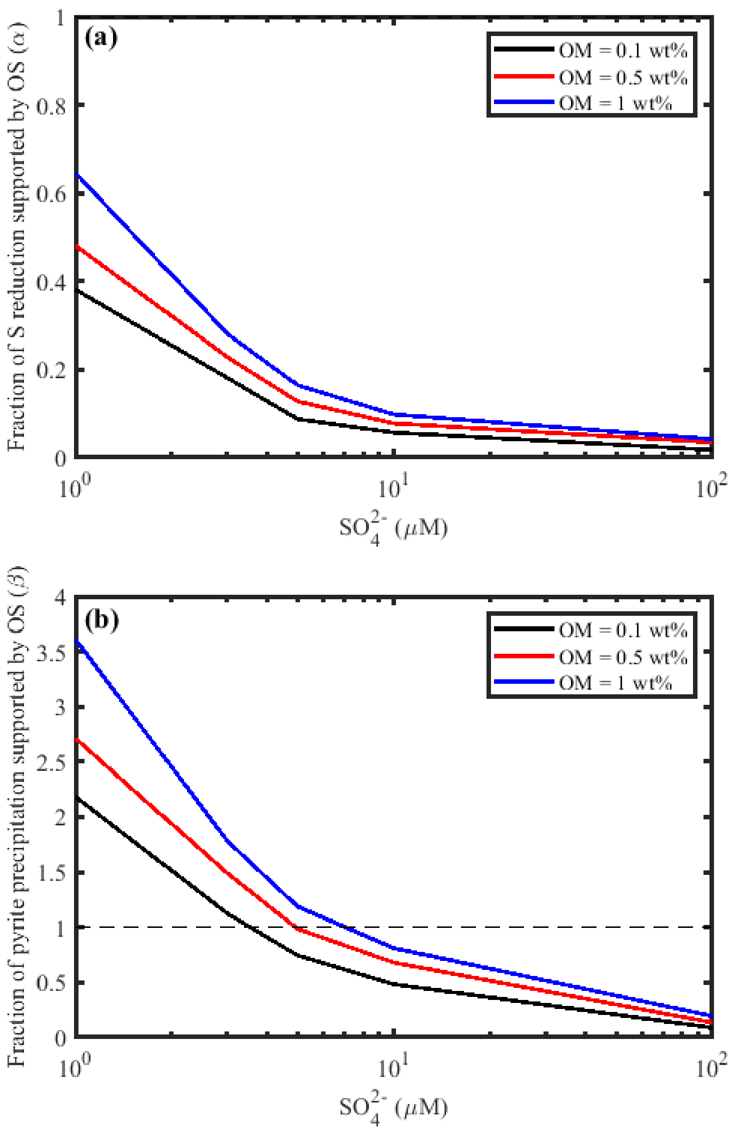

Supplementary Figure 3. Sensitivity of α **(a)** and β **(b)** to organic matter (OM) availability, under anoxic conditions.

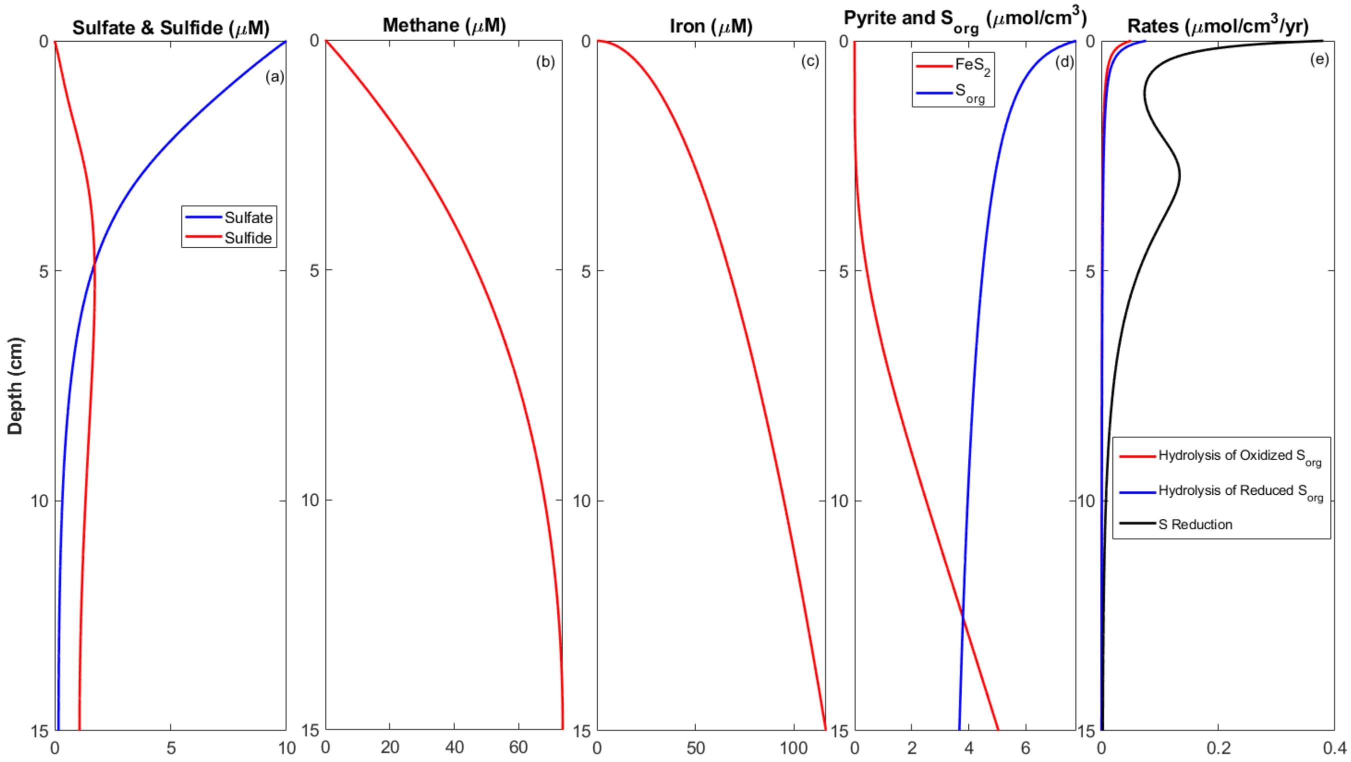

Supplementary Figure 4. Typical sediment distributions of the concentrations of **(a)** sulfate, hydrogen sulfide, **(b)** methane, and **(c)** dissolved iron (II), **(d)** the concentrations of pyrite and organic sulfur, and **(e)** rates of oxidized and reduced organic sulfur hydrolysis/mineralization and S reduction.

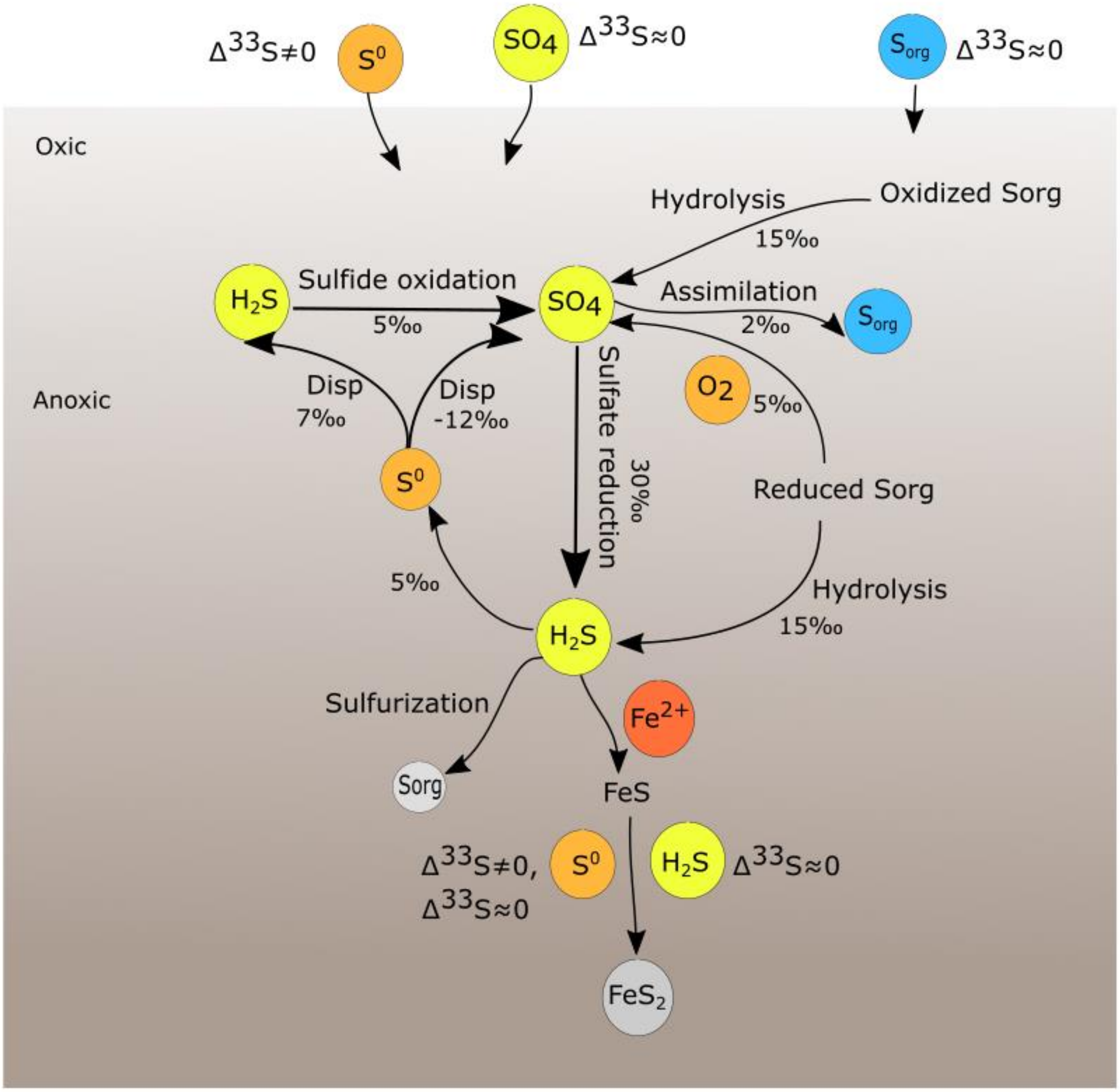

Supplementary Figure 5. Sediment sulfur cycle with contribution from organic sulfur hydrolysis. Isotopic fractionations during organic sulfur hydrolysis that are smaller than those during sulfate reduction can produce sedimentary sulfide (FeS_2) with respectively smaller $\Delta^{34}S_{FeS2}$ values (See Methods).

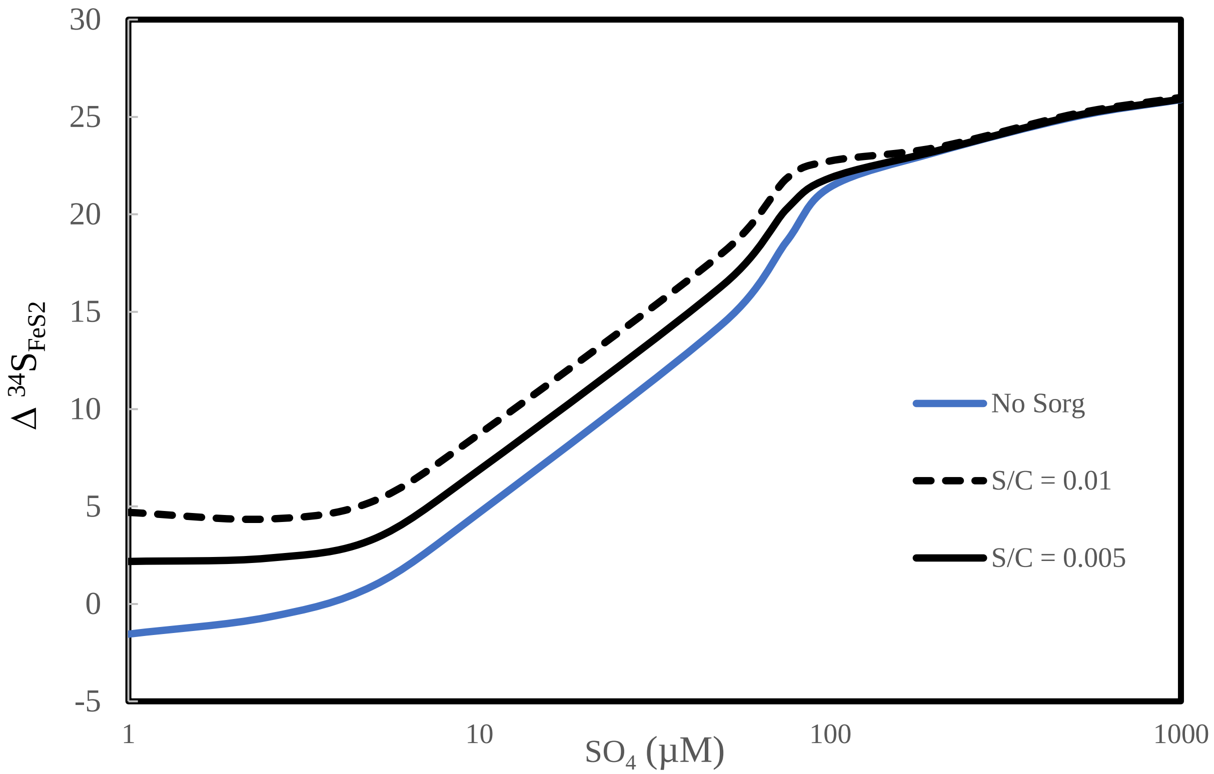

Supplementary Figure 6. Isotopic effects of organic sulfur on $\Delta^{34}S_{FeS2}$. Isotopic fractionation du ring organic sulfur hydrolysis generates isotopically lighter pyrite, increasing $\Delta^{34}S_{FeS2}$. Different lines correspond to different S:C ratios.

Supplementary Tables

Supplementary Table 1. Reactions included in the model

**Reactions**	**Rate**
$CH_2O + O_2 \Rightarrow CO_2 + H_2O$	R_{OX}
$CH_2O + 4\ FeOOH_{(s)} + 7CO_2 + H_2O \Rightarrow 4Fe^{2+} + 8\ HCO_3^{-}$	R_{FeOOH}
$SO_4^{2-} + 2CH_2O \Rightarrow H_2S + 2HCO_3$	R_{SR}
$H_2S + 2O_2 + 2HCO_3^{-} \Rightarrow SO_4^{2-} + 2CO_2 + 2H_2O$	R_{SOX}
$R\text{-}O\text{-}SO_3H + H_2O \Rightarrow HSO_4^{2-} + R\text{-}OH_2$	R_{PSO4}
$R\text{-}SH + H_2O \Rightarrow H_2S + R\text{-}OH$	R_{PH2S}
$Fe^{2+} + HS^{-} \Rightarrow FeS(s) + H^{+}$	R_{FeS}
$2FeOOH_{(s)} + 3H_2S_{(aq)} => S^0_{(s)} + 2FeS_{(s)} + 4H_2O$	R_{S0}
$4\ S^0_{(s)} + 4H_2O => SO_4^{2-} + 3HS^{-} + 5H^{+}$	R_{Disp}
$FeS + S^0 => FeS_2$	R_{FeS+S}
$FeS + H_2S => FeS_2 + H_2$	R_{FeS+HS}
$2CH_2O => CH_4 + CO_2$	R_{CH4}
$CH_4 + 2O_2 => CO_2 + 2H_2O$	R_{CH4_O2}
$SO_4^{2-} + CH_4 => H_2S + CO_2 + H_2O$	R_{CH4_SO4}
$SO_4^{2-} \Rightarrow R\text{-}SH$	R_{Assim}

Supplementary Table 2. Kinetics of the reactions included in the model. K_{FeS}, K_{H2S}, and k_{Disp} are the equilibrium constants for FeS precipitation, acidity constant for H_2S, and rate constant of S^0 disproportionation, respectively.

**Reaction**	**Rate expression**
Sulfide Oxidation	$R_{SOX} = k_{SOX}[O_2][H_2S]$
Sulfate production from R-O-SO_3H	$R_{PSO4} = f_{SO_4} * \frac{S}{C} V_m$; Where S/C is the sulfur to carbon molar ratio; V_m = organic carbon mineralization rate.
Sulfide production from R-SH	$R_{PH2S} = f_{H_2S} * \frac{S}{C} V_m$
Aerobic Respiration	$R_{OX} = \frac{V_m[O_2]}{K_i + [O_2]}$
Iron Reduction	$R_{FeOOH} = \frac{4V_m[FeOOH]}{K_{FeOOH} + [FeOOH]} \frac{K_i}{K_i + [O_2]}$
Sulfide Precipitation	$R_{FeS} = k_{FeS}(\frac{\Omega}{K_{FeS}} - 1)$<br>$\Omega = \frac{[Fe^{2+}][HS^-]}{[H^+]}$<br>$[HS^-] = \frac{[H_2S]}{1 + [H^+]K_{H_2S}}$
Sulfate Reduction	$R_{SR} = \frac{0.5V_m[SO_4^{2-}]}{K_m + [SO_4^{2-}]} \frac{K_i}{K_i + [O_2]} \frac{K_{FeOOH}}{K_{FeOOH} + [FeOOH]}$
Elemental Sulfur Formation	$R_{S0} = k_{S0}[FeOOH][H_2S]$
Aerobic methane oxidation	$R_{CH4-O2} = k_{CH4-O2}[CH4] \frac{[O_2]}{K_i + [O_2]}$
Anaerobic methane oxidation	$R_{CH4-SO4} = k_{CH4-SO4}[CH4] \frac{[SO_4^{2-}]}{K_{AOM} + [SO_4^{2-}]}$
Methane Production	$R_{CH_4} = 0.5V_m \frac{K_i}{K_i + [O_2]} \frac{K_{FeOOH}}{K_{FeOOH} + [FeOOH]} \frac{K_m}{K_m + [SO_4^{2-}]}$
Elemental Sulfur Disproportionation	$R_{Disp} = k_{Disp}[S^0](1 - \frac{[H_2S]}{[H_2S]_{stop}})$ for $[H_2S] < [H_2S]_{stop}$<br>0 for $[H_2S] \geq [H_2S]_{stop}$
FeS_2 precipitation with S^0	$R_{FeS+S} = k_{FeS+S}[FeS][S^0]$
FeS_2 precipitation with H_2S	$R_{FeS+HS} = k_{FeS+HS}[FeS][H_2S]$

Supplementary Table 3. Parameters in the reactive transport model. Asterisk (*) indicates parameters that were randomly varied in the sensitivity analysis within their specified expected ranges. SWI = sediment-water interface.

Parameter	Symbol	Value	Unit	Expected Range	Ref.
*Diffusion coefficient of sulfate	D_{SO4}	300	$cm^2\ yr^{-1}$	200-400	1
*Diffusion coefficient of sulfide	D_{H2S}	500	$cm^2\ yr^{-1}$	400-600	2
*Diffusion coefficient of oxygen	D_{O2}	500	$cm^2\ yr^{-1}$	300-500	2
*Sulfide oxidation rate constant	k_{OXD}	160	$uM^{-1}yr^{-1}$	100-1000	3
*FeS precipitation rate constant	k_{FeS}	10^{-5}	$mol\ g^{-1}\ yr^{-1}$	10^{-6}-10^{-5}	3
FeS equilibrium constant	K_{FeS}	10^{-4}	M	-	4
*Sulfur-to-carbon ratio in organic matter	S:C	0.005	mol/mol	0.003-0.01	5, 6
*Fraction of oxidized organic sulfur	f_{SO4}	0.4	-	0.2-0.6	-
*Fraction of reduced organic sulfur	f_{H2S}	0.6	-	0.4-0.8	-
Acid-base equilibrium constant for H_2S	K_{H2S}	$2.48x10^{-7}$	-	-	4
*S^0 formation rate constant	k_{S0}	$8x10^{-3}$	$\mu M^{-1}yr^{-1}$	10^{-4}-10^{-2}	7
*S^0 disproportionation rate constant	k_{Disp}	0.001	yr^{-1}	10^{-4}-10^{-2}	8
Threshold H_2S concentration for disproportionation	$[H_2S]_{stop}$	10	mM	-	8
*Monod constant for SO_4^{2-} reduction	K_m	20	uM	5-77	3
*Monod constant for O_2 (inhibition constant)	K_i	1	µM	0.5 – 2	3
Monod constant for FeOOH reduction	K_{FeOOH}	20	µmol/g	15-30	3
*Aerobic methane oxidation rate constant	k_{CH4-O2}	10	$year^{-1}$	1-100	9
*Anaerobic methane oxidation rate constant	$k_{CH4-SO4}$	0.056	$year^{-1}$	0.0056-0.56	10, 11
Monod constant for AOM	K_{AOM}	100	µM	100-500	11
*Sulfate at SWI (sediment water interface)	$[SO_4^{2-}]$	10	µM	1-1000	-
*Methane at SWI (sediment water interface)	$[CH_4]$	0	µM	0-200	-
Sulfide concentration at SWI	$[H_2S]$	0	µM	-	-
*Oxygen concentration at SWI	$[O_2]$	0	µM	0-30	-
*Fe^{2+} at SWI	$[Fe^{2+}]$	0	µM	0-10	-
*Fe(III) concentration at OPD	$[Fe(OH)_3]_{OPD}$	5	mg/g	0-25	-
Density of dry sediment	ρ_s	2.65	g/cm^3	-	-

Grid size	dz	6.67×10^{-3}	cm	-	-
Flux of S^0 at SWI	$Flux_{S0}$	0.4	μmol/cm^2/yr	0.1-1	
*pH	pH	6	-	6-7	12
*Organic carbon content	OC	0.5	wt%	0.1-1	
*Burial velocity	V_{burial}	0.1	cm yr^{-1}	0.05-0.2	-
Initial age of organic carbon	Age_{init}	1	yr	1-50	13
Sediment depth	L	20	cm		

Supplementary References

1. Canfield D. E. Models of oxic respiration, denitrification and sulfate reduction in zones of coastal upwelling. *Geochim. Cosmochim. Acta* **70**, 5753–5765 (2006).

2. Burdige D. J. Geochemistry of marine sediments., Princeton University Press, Princeton (2006).

3. Katsev S., Rancourt D. G. and L'Heureux I. dSED: A database tool for modeling sediment early diagenesis. *Comput. Geosci.* **30** (2004).

4. Dyrssen D. and Kremling K. Increasing hydrogen sulfide concentration and trace metal behavior in the anoxic Baltic waters. *Mar. Chem.* **30**, 193–204 (1990).

5. Urban N. ., Ernst K. and Bernasconi S. Addition of sulfur to organic matter during early diagenesis of lake sediments. *Geochim. Cosmochim. Acta* **63**, 837–853 (1999).

6. Ho T.-Y., Quigg A., Finkel Z. V., Milligan A. J., Wyman K., Falkowski P. G. and Morel F. M. M. The elemental composition of some marine phytoplankton. *J. Phycol.* **39**, 1145–1159 (2003).

7. Van Cappellen P. and Wang Y. Cycling of iron and manganese in surface sediments; a general theory for the coupled transport and reaction of carbon, oxygen, nitrogen, sulfur, iron, and. *Am. J. Sci.* **296**, 197–243 (1996).

8. Jourabchi P. New developments in early diagenetic modeling: pH distributions, calcite dissolution and compaction. Utrecht University (2007).

9. Van Bodegom, P., Stams, F., Mollema, L., Boeke, S., & Leffelaar, P. Methane oxidation and the competition for oxygen in the rice rhizosphere. Appl. Environ. Microbiol. 67, 3586-3597 (2001).

10. Ingvorsen K., Jørgensen B. B., Kinetics of sulfate uptake by freshwater and marine species of Desulfovibrio. Arch. Microbiol. **139**, 61-66 (1984).

11. Olson, S. L., Reinhard, C. T., & Lyons, T. W. Limited role for methane in the mid-Proterozoic greenhouse. *PNAS*, **113,** 11447-11452 (2016).

12. Halevy I. and Bachan A. The geologic history of seawater pH. *Science* **355**, 1069–1071 (2017).

13. Fakhraee, M., Crowe, S. A. & Katsev, S. Sedimentary sulfur isotopes and Neoarchean ocean oxygenation. *Sci. Adv.* **4**, e1701835 (2018).
